# A Workflow towards the Reproducible Identification and Quantitation of Protein Carbonylation Sites in Human Plasma

**DOI:** 10.3390/antiox10030369

**Published:** 2021-03-01

**Authors:** Juan Camilo Rojas Echeverri, Sanja Milkovska-Stamenova, Ralf Hoffmann

**Affiliations:** 1 Institute of Bioanalytical Chemistry, Faculty of Chemistry and Mineralogy, Universität Leipzig, 04103 Leipzig, Germany; juan_camilo.rojas_echeverri@uni-leipzig.de (J.C.R.E.); sanja.milkovska@uni-leipzig.de (S.M.-S.); 2 Center for Biotechnology and Biomedicine, Universität Leipzig, 04103 Leipzig, Germany

**Keywords:** protein carbonylation, human plasma, aldehyde reactive probe (ARP), biotin-avidin affinity, LC-MS/MS

## Abstract

Protein carbonylation, a marker of excessive oxidative stress, has been studied in the context of multiple human diseases related to oxidative stress. The variety of post-translational carbonyl modifications (carbonyl PTMs) and their low concentrations in plasma challenge their reproducible identification and quantitation. However, carbonyl-specific biotinylated derivatization tags (e.g., aldehyde reactive probe, ARP) allow for targeting carbonyl PTMs by enriching proteins and peptides carrying these modifications. In this study, an oxidized human serum albumin protein model (OxHSA) and plasma from a healthy donor were derivatized with ARP, digested with trypsin, and enriched using biotin-avidin affinity chromatography prior to nano reversed-phase chromatography coupled online to electrospray ionization tandem mass spectrometry with travelling wave ion mobility spectrometry (nRPC-ESI-MS/MS-TWIMS). The presented workflow addresses several analytical challenges by using ARP-specific fragment ions to reliably identify ARP peptides. Furthermore, the reproducible recovery and relative quantitation of ARP peptides were validated. Human serum albumin (HSA) in plasma was heavily modified by a variety of direct amino acid oxidation products and adducts from reactive carbonyl species (RCS), with most RCS modifications being detected in six hotspots, i.e., Lys10, Lys190, Lys199, Lys281, Lys432, and Lys525 of mature HSA.

## 1. Introduction

Reduction and oxidation are essential reactions for energy production and regulation of normal homeostasis in all living organisms. However, this physiological oxidative eustress can be disrupted when oxidative pathways are excessively activated [1]. This oxidative distress can trigger irreversible, oxidative post-translational modifications (OxPTMs), such as electrophilic additions of lipid peroxidation products (LPPs) [2,3,4], advanced glycation end products (AGEs) [5], and amino acid oxidation by metal-catalyzed oxidation (MCO) or other oxidants [6]. These modifications and oxidative distress have been linked to several human pathologies (e.g., diabetes, cardiovascular diseases, neurological disorders, and autoimmune disorders) and have been proposed as potential biomarkers of oxidative distress. Thus, analytical methods allowing qualitative and quantitative analyses in blood are required for diagnosis and prognosis.

The complexity of the blood proteome and its OxPTMs demands high-performance liquid chromatography (HPLC) techniques coupled online to high-resolution mass spectrometers (LC-MS/MS) as a prerequisite of biomarker discovery. Unfortunately, the detection of these modifications has proven very challenging due to the low modification degrees, the interfering chemical background, and the large diversity of OxPTMs that can occur at any residue. However, a subset of OxPTMs containing so-called reactive carbonyls (i.e., aldehydes or ketones) has gained much interest as biomarkers of protein oxidation [7,8]. They can be selectively derivatized at aldehyde and keto groups allowing their selective detection and enrichment by affinity-chromatography. Most commercially available derivatization reagents rely on a biotin tag for enrichment and a hydroxylamine or a hydrazine as reactive group yielding rather stable oxime adducts and hydrazone analogues, respectively [9]. Due to the good stability of oximes, *O*-(biotinylcarbazoylmethyl)hydroxylamine (also known as aldehyde reactive probe or ARP) is commonly used in LC-MS/MS-based studies for the efficient derivatization of carbonylated peptides and proteins [10,11], targeting carbonylation sites in vitro [11,12,13,14,15], in animal models [16], and in human samples [17]. These bottom-up proteomics approaches rely on the enrichment either of derivatized proteins followed by enzymatic digestion or of derivatized peptides after digestion. A recent study comparing both approaches [15] showed that enrichment at the protein level allowed for proposing many carbonylated proteins, but modification sites were only scarcely detected. Enrichment at the peptide level proposed less proteins but identified more carbonylation sites per protein, most likely due to the better depletion of non-derivatized peptides.

Identification of derivatized carbonylation sites by mass spectrometry is challenging. Collision-induced dissociation (CID) of derivatized peptides produces intense signals specific to the derivatization tag (e.g., reporter ions) decreasing the confidence scores reported by some software tools, although they can also improve the identification accuracy of derivatized peptides [11,12,13,14,18,19]. Thus, it appears favorable to remove the intense reporter ion signals from the acquired mass spectra [16,20]. It should be noted that the mentioned studies relied mainly on MASCOT (Matrix Science) and its scoring approach pitfalls, described in detail by Slade et al. [16], may not apply to other search engines.

Many studies relied on LC-MS/MS to identify carbonylated proteins and map the corresponding modification sites in human plasma [17,20,21,22]. However, to the best of our knowledge, reproducible quantitation of carbonylation sites and more generally OxPTMs in human plasma—a prerequisite for testing their biomarker potential—has not been reported to date. This work addresses some analytical challenges for quantitation of OxPTM sites, providing an improved workflow for sample preparation, data acquisition, data validation, and identification and quantitation of carbonylation sites in human plasma proteins.

## 2. Materials and Methods

### 2.1. Materials

Materials were obtained from the following suppliers. Advansta Inc. (San Jose, CA, USA): AdvanWash buffer for washing and protein-free AdvanBlot-PF blocking buffer; AppliChem GmbH (Darmstadt, Germany): disodium ethylenediaminetetraacetate dehydrate (EDTA, 99%), iodoacetamide (IAA, ≥99%), and tris(hydroxymethyl)aminomethane (Tris, ≥99.9%); BioRad Laboratories GmbH (Munich, Germany): Immun-Blot low-fluorescence polyvinylidene difluoride (LF PVDF) membranes, Oriole fluorescent gel stain, Precision Plus Protein standards, Trans-Blot Turbo mini-size transfer stacks, and Trans-Blot Turbo transfer buffer; Biosolve GmbH (Valkenswaard, The Netherlands): acetonitrile (ultra-high performance liquid chromatography-mass spectrometry (uHPLC-MS) grade, ≥99.97%), formic acid (uHPLC-MS-grade, ≥99%), and methanol (uHPLC-MS-grade, ≥99.98%); Carl Roth GmbH (Karlsruhe, Germany): dithiothreitol (DTT, ≥99%), glycerol (≥99.5%), hydrogen peroxide (30%), sodium dodecyl sulfate (SDS, ≥99.5%), and urea (≥99.5% p.a.); Dojindo Laboratories (Kumamoto, Japan): aldehyde reactive probe (ARP); Honeywell Riedel-de Haën (Steinheim, Germany): bromophenol blue sodium salt; Merck KGaA (Darmstadt, Germany): Microcon-10 kDa regenerated cellulose centrifugal filters; ^NH^Dyeagnostics (Halle, Germany): Immuno Blue Western Blotting substrate; Pierce Biotechnology (Rockford, IL, USA): biotinylated bovine serum albumin (Biot-BSA), 50% slurry of immobilized monomeric avidin on agarose beads, and spin columns; SERVA Electrophoresis GmbH (Heidelberg, Germany): acrylamide/bis solution (37.5:1, 30% *w*/*v*, 2.6% C), ammonium persulfate (analytical grade), Coomassie Brilliant Blue G-250, human serum albumin fraction V, and modified sequencing grade trypsin from porcine pancreas; Sigma-Aldrich Chemie GmbH (Steinheim, Germany): ammonium bicarbonate (≥99.5%), aniline (≥99.5%), β-mercaptoethanol (≥99%), copper sulfate pentahydrate (99%), ExtrAvidin-Peroxidase, hydrochloric acid (HCl, 36.5–38%), (+)-sodium-l-ascorbate (≥99.0%), sodium deoxycholate (≥97%), sodium chloride (≥NaCl, 99.5%), sodium phosphate dibasic dodecahydrate (≥99.0%), sodium phosphate monobasic (≥99.0%), tris-(2-carboxyethyl) phosphine (TCEP, ≥98%), and TPCK-treated trypsin from bovine pancreas; WATERS (Milford, OH, USA): Oasis HLB 10 mg solid phase extraction (SPE) cartridges.

Water was purified in-house (resistance >18 MΩ cm^−1^; total organic content <10 ppb) on a PureLab Ultra Analytic System (ELGA Lab Water, Celle, Germany).

### 2.2. Human Plasma Collection

Human plasma was obtained from a healthy male volunteer who provided a written informed consent. Blood was collected in 3 aliquots of 9.0 mL in S-Monovette K3 EDTA tubes (SARSTEDT, Nümbrecht, Germany) after 16 h of fasting. The samples were centrifuged at 2000× *g* first for 5 min and then again 10 min (4 °C). Plasma aliquots (100 µL) were stored at −80 °C.

### 2.3. Derivatization of Reactive Carbonyl Groups

The protein content in human plasma was determined using the microBradford assay. A protein aliquot (200 µg) was diluted with ammonium bicarbonate (25 mmol/L, pH 8), aniline (0.1 mol/L) in ammonium bicarbonate (25 mmol/L), or formic acid 1% (*v*/*v*, pH 2) to a final volume of 500 µL; transferred to a pre-conditioned 10 kDa molecular weight cut-off (MWCO) ultrafiltration unit; and centrifuged (14,000× *g*, 30 min, 25 °C). More reaction solvent (500 µL) was added to the cartridge and centrifuged again using the same conditions. This step was repeated twice. An aqueous solution of ARP (25 mmol/L) was diluted with the reaction solution to achieve final concentrations of 0.5 or 5 mmol/L in 200 µL. The samples were incubated overnight (25 °C, dark, 300 rpm). Ammonium bicarbonate (25 mmol/L, 500 µL) was added and centrifuged (same conditions as before). This step was repeated twice. The cartridges were inverted, and the protein solutions were collected in pre-weighed collection tubes by centrifugation (1000× *g*, 3 min, 25 °C). Protein concentrations were determined by a microBradford assay. Six samples were prepared in parallel at 2 ARP concentrations.

Biot-BSA (20 ng) derivatized or underivatized plasma proteins (negative control, 1 µg each) were mixed with Laemmli sample buffer (Tris/HCl (62.5 mmol/L, pH 6.8), glycerol (20%, (*v*/*v*)), SDS (2% *w*/*v*), β-mercaptoethanol (5% (*v*/*v*)), bromophenol blue (0.025% *w*/*v*)), incubated (4 min, 95 °C), and separated by SDS polyacrylamide gel electrophoresis (SDS-PAGE, 12% T, 1 mm, 200 V; BioRad mini protean III cell; BioRad Laboratories GmbH, München, Germany). Proteins were visualized using Oriole fluorescent stain (λ_exc_ = 270 nm, λ_em_ = 604 nm) and subsequent colloidal Coomassie Brilliant Blue G-250 [23]. Images were taken on a Gel Doc EZ Imager ChemiDoc system (Bio-Rad Laboratories). Alternatively, proteins were blotted onto a LF PVDF membrane using a Trans-Blot Turbo transfer cell (Bio-Rad Laboratories; 25 V, 1.3 A, room temperature (RT), 10 min). The membrane was washed with water and blocked overnight with AdvanBlot-PF blocking solution (20 mL, RT); then, extravidin-peroxidase solution (1 µL) was added, and the membrane was incubated at RT for 1 h and washed with AdvanWash solution (20 mL, 3×, 5 min). The membrane was incubated with Immuno Blue HRP-Substrate (10 min) and washed with AdvanWash solution (20 mL, 2 min) twice. The fluorescence was recorded on a ChemiDoc MP CCD camera system (Bio-Rad Laboratories) and analyzed by integrating the intensity of the whole protein lane (ImageLab 6.0.1).

#### 2.3.1. Oxidized Human Serum Albumin as a Protein Model

Stock solutions of human serum albumin (HSA, 1 µg/µL corresponding to 15 µmol/L) were oxidized in aqueous ammonium bicarbonate (25 mmol/L) containing copper sulfate (0.3 mmol/L), (+)-sodium-L-ascorbate (0.6 mmol/L), and hydrogen peroxide (1 mmol/L). The mixture was sonicated for 15 min, incubated (1 h, 300 rpm, 37 °C), and quenched by adding EDTA to a final concentration of 2.5 mmol/L. An aliquot containing 200 µg of oxidized HSA (OxHSA) was diluted with formic acid (500 µL, 1% (*v*/*v*)) and transferred to a pre-conditioned 10 kDa MWCO filter. Samples were centrifuged (14,000× *g*, 30 min, 25 °C), formic acid was added (500 µL, 1% (*v*/*v*)), and the samples were centrifuged again. This step was repeated twice. Finally, ARP (25 mmol/L in water) was added to achieve a final concentration of 5 mmol/L in formic acid (200 µL, 1% (*v*/*v*)), and the samples were incubated overnight in darkness with gentle shaking.

#### 2.3.2. Plasma Samples

Aliquots of plasma (protein contents of 0.2 or 2 mg) were diluted with formic acid (1% (*v*/*v*)) to a final volume of 500 µL and transferred to a pre-conditioned 10 kDa MWCO ultrafiltration unit. Solvent exchange and derivatization followed the protocol described above for OxHSA.

### 2.4. Protein Digestion

#### 2.4.1. Filter-Aided Sample Preparation (FASP)

Plasma proteins were digested by the FASP protocol [24]. Briefly, a urea solution (8 mol/L urea in 0.1 mol/L Tris-HCl; pH 8.5) was added to the ultrafiltration units containing derivatized proteins (0.2 or 2 mg) to obtain a total volume of 500 µL and centrifuged for 50 min (25 min for 0.2 mg sample). This step was repeated once followed by disulfide reduction using DTT (12.5 µL, 500 mmol/L, 1 h, 37 °C, 550 rpm). The samples were centrifuged (20 min, 14,000× *g*, 25 °C), and urea solution was added (200 µL); then, the samples were centrifuged again (20 min, 14,000× *g*, 25 °C) and alkylated with IAA (100 µL, 50 mmol/L, 20 min, darkness, RT). The samples were centrifuged (10 min, 14,000× *g*, 25 °C); then, urea solution (100 µL) was added, and the samples were centrifuged again for 50 min (14,000× *g*, 25 °C; 15 min for 0.2 mg samples). Ammonium bicarbonate solution (100 µL, 0.1 mol/L) was added and the samples were centrifuged for 50 min (14,000× *g*, 25 °C; 15 min for 0.2 mg sample). The addition of ammonium bicarbonate solution and centrifugation was repeated twice. Trypsin (80 µg in 32 µL) was added to the ultrafiltration unit (8 µg for the 0.2 mg samples) and digested overnight (25:1 protein/enzyme ratio, wet chamber, 37 °C). The collection tubes were exchanged, and the digest was centrifuged (10 min, 14,000× *g*, 25 °C). Ammonium bicarbonate solution (50 µL) was added, and the samples were again centrifuged (15 min, 50 µL). The addition of ammonium bicarbonate solution and centrifugation was repeated once before the filtrate was dried under vacuum. When necessary, digests were dissolved in aqueous acetonitrile (30% (*v*/*v*)) containing formic acid (0.1% (*v*/*v*)) to take an aliquot corresponding to 10 µg of digest for analysis prior to affinity chromatography. Finally, samples were dried again under vacuum and stored at −20 °C.

#### 2.4.2. In-Solution Digest

Aliquots of plasma (*n* = 8) corresponding to 2.5 mg protein were diluted with aqueous ammonium bicarbonate (25 mmol/L, 2.5 mL) to be digested in solution [17]. Proteins were denatured and reduced by addition of aqueous sodium deoxycholate (312.5 µL, 10% *w*/*v*) and TCEP (312.5 µL, 50 mmol/L, 30 min, 60 °C, 550 rpm) and alkylated with IAA (345 µL, 0.1 mol/L, 30 min, in darkness, 37 °C, 550 rpm). Excess of IAA was quenched with DTT (385 µL, 0.1 mol/L, 30 min, 37 °C, 550 rpm) and trypsin (125 µL, 0.4 µg/µL) added. After incubation overnight (50:1 protein/enzyme ratio, 37 °C, 550 rpm), formic acid (20 µL, 0.5% (*v*/*v*) final concentration) was added, and the solution split in half and centrifuged (30 min, 14,000× *g*, 4 °C). Both supernatants were pooled, loaded on a pre-conditioned (1 mL methanol, 2×1 mL aqueous formic acid (0.1% (*v*/*v*)) Oasis HLB SPE cartridge (1 mL, 10 mg), washed (3×1 mL aqueous formic acid (0.1% (*v*/*v*)), and eluted with *aq.* acetonitrile (0.6 mL, 70%, (*v*/*v*)) containing formic acid (0.1% (*v*/*v*)). The eluates were dried under vacuum and stored at −20 °C.

### 2.5. Avidin Affinity Chromatography

Mini-spin columns were packed with monomeric avidin agarose beads (50% slurry, 200 µL) and washed with phosphate buffer (1.5 mL, 10 mmol/L phosphate; pH 7.4) and phosphate-buffered saline (PBS, 2 mL, 20 mmol/L phosphate, 300 mmol/L NaCl; pH 7.4). Dried ARP-labeled samples were reconstituted in PBS (see Section 2.6 for details) and loaded on the column with gravity flow. The columns were washed with PBS (1 mL), phosphate buffer (1 mL), ammonium bicarbonate (50 mmol/L) in *aq*. methanol (20% (*v*/*v*), 2 mL), and water (1 mL). The peptides were eluted with *aq.* acetonitrile (500 µL, 30% (*v*/*v*)) containing formic acid (0.4% (*v*/*v*)) and diluted twofold with water, loaded in aliquots on a pre-conditioned 10 kDa MWCO ultrafiltration unit, and centrifuged (25 min, 14,000× *g*, 25 °C). The filtrates were pooled in a 2 mL Eppendorf tube, dried under vacuum, and stored at −20 °C. Just before MS, the samples were dissolved in 100 µL of *aq.* acetonitrile (3% (*v*/*v*)) containing formic acid (0.1% (*v*/*v*)).

### 2.6. Workflow Performance Evaluation

Enrichment reproducibility and recovery were assessed by spiking ARP-derivatized OxHSA (ARP-OxHSA; 64.4 µg based on the initial protein content) digested by FASP into ARP-derivatized plasma (ARP-Plasma; 580 µg) digested by FASP as well. An aliquot (10 µg protein) of this ARP-Plasma/ARP-OxHSA mixture was stored (non-enriched control) while the rest was dried under vacuum and reconstituted with PBS (2 g/L). Three aliquots of the mixture (95 µL) and one aliquot containing only ARP-Plasma digest (190 µg) were enriched independently by avidin affinity chromatography (triplicate) and subjected to ultrafiltration in parallel (25 min, 14,000× *g*, 25 °C; Figure S1A).

The effects of matrix interferences on the recovery of ARP-derivatized peptides were evaluated by spiking an in-solution digest of plasma with ARP-OxHSA digested by FASP (64.4 µg) at protein ratios of 9:1, 49:1, and 249:1 (Plasma:ARP-OxHSA). Each mix was diluted with PBS to achieve a concentration of 0.039 µg/µL of ARP-OxHSA spike. Three aliquots of each ratio mix (500 µL each) were processed in parallel (Figure S1B).

The reproducibility and upscaling efficiency of the method was assessed for protein quantities of 0.2 and 2.0 mg using 3 different human plasma aliquots for each protein content. The 6 samples were derivatized in parallel with ARP under acidic conditions as described above, and were subsequently digested with trypsin using the outlined FASP procedure. After digestion and drying, each sample was dissolved in aqueous acetonitrile (30% (*v*/*v*)) containing formic acid (0.1% (*v*/*v*)), and an aliquot from each sample (10 µg protein, non-enriched controls) was stored at −20 °C. The remaining part of each sample was dried under vacuum and dissolved in 0.1 mL (0.2 mg ARP-Plasma digest) or 1 mL (2 mg ARP-Plasma digest) of PBS prior to enrichment. The 6 digests were enriched by affinity chromatography in parallel as described above (see Figure S1C for schematic representation).

### 2.7. Mass Spectrometry Acquisition (nRPC-ESI-MS/MS-TWIMS)

The affinity-enriched peptides were separated on a nanoACQUITY Ultra Performance LC™ (Waters Corp., Manchester, UK) coupled online to a Q-TOF SYNAPT G2-S*i* instrument (Waters Corp., Manchester, UK) using digests of non-enriched samples (35 ng; 70 ng for ARP-Plasma/ARP-OxHSA samples) and eluted fractions of the affinity chromatography (5.8% on column). Peptides were trapped (nanoACQUITY Symmetry C18-column, internal diameter (ID) 180 µm, length 2 cm, particle diameter 5 µm) at a flow rate of 5 µL/min (3% (*v*/*v*) aqueous acetonitrile containing 0.1% (*v*/*v*) formic acid, 6 min). Separation relied on a BEH 130 column (C18-phase, ID 75 µm, length 10 cm, particle diameter 1.7 µm; 35 °C) and a flow rate of 0.3 µL/min using a segmented linear gradient from 3% to 30% (27 min), 30% to 35% (10 min), 35% to 40% (5 min), and 40% to 85% (4 min) aqueous acetonitrile (*v*/*v*) containing 0.1% (*v*/*v*) formic acid. The nanospray used a PicoTip Emmitter (New Objective, Littleton, US) at a spray voltage of 3 kV, sampling cone of 30 V, source offset of 80 V, source temperature of 100 °C, cone gas flow of 20 L/h, and nanoflow gas pressure of 0.2 bar. Mass spectra were recorded in positive ion mode using a high-definition data-dependent acquisition approach (HD-DDA) where both precursor and fragment ions were separated by travelling wave ion mobility spectrometry (T-Wave IMS, TWIMS). Full-scan MS and MS/MS spectra (50–5000 *m*/*z*) were acquired in resolution mode (R = 20,000 at *m*/*z* 400; FWHM) using a MS scan of 0.2 s. MS/MS scans were triggered for signal intensities above 1000 counts and acquired once up to a total ion current (TIC) threshold of 100,000 counts for a maximum time of 0.4 s. Fragmentation was induced in the trapping region of the ion mobility cell utilizing an *m*/*z*-dependent collision energy ramp from 6/9 V (50 *m*/*z*, start/end) up to 147/183 (5000 *m*/*z*, start/end). Tandem mass spectra were triggered for the 5 most intense signals using a dynamic exclusion window of ±250 mDa for 6 s using reported TWIMS settings [25], i.e., a full cycle ramped wave velocity of 2500 to 400 m/s (start to end), trapping before IMS for 500 µs with 15 V, 0 V extraction, and IMS delay of 1000 µs after trap release. Finally, an enhanced duty cycle approach was implemented [25], which significantly increases the sensitivity of singly charged fragment ions by synchronizing their ion mobility to the orthogonal TOF pusher and detector. The synchronization was performed using MassLynx V4.2 SCN983 and DriftScope v2.9 on the basis of the IMS-*m*/*z* profile of singly charged fragment ions resulting from the doubly protonated Glu-fibrinopeptide-B, which was infused through the reference sprayer. Reference scans were acquired every 30 s for post-acquisition lock mass recalibration considering the signal at *m*/*z* 785.843.

### 2.8. Data Analysis

#### 2.8.1. Database Search

LC-MS/MS raw files were imported into PEAKS Studio 10.5 (Bioinformatics Solutions, Waterloo, ON, Canada) and corrected for the lock mass with PEAKS built-in loader using the signal at *m*/*z* 785.843 considering an error tolerance of 0.5 Da. Tandem mass spectra were processed by a DeNovo procedure considering cysteine carbamidomethylation (+57.022 Da) and methionine oxidation (+15.9949 Da) as variable modifications. The results were searched against Human Swissprot protein database (accessed on 4 April 2019) and the common repository of adventitious proteins (cRAP) contaminants database (https://www.thegpm.org/crap) using a PEAKS DB procedure (first pass) considering the same 2 variable modifications. Peptides with at least 1 terminal trypsin cleavage site and up to 3 missed tryptic cleavage sites were considered for further data processing. All non-assigned tandem mass spectra were processed with a PEAKS PTM procedure (second pass) considering all targeted modifications (Table S1) as variable modifications and using the error and peptide specificity settings above for the PEAKS DB search. PEAKS PTM greatly reduces the search space and time by only considering proteins identified by the PEAKS DB search. However, this approach is not recommended for peptide samples that have been affinity enriched. Therefore, tandem mass spectra recorded for samples containing derivatized plasma proteins were processed with an additional PEAKS DB (first pass) search considering all targeted modifications to also cover proteins represented only by derivatized and enriched peptides. All searches were repeated with carbamidomethylation as a fixed modification. Datasets were filtered in PEAKS Studio 10.5 by setting a 5% false discovery rate (FDR) at the peptide level (Table S2). The peptide spectrum matches (PSM) identification results were exported as text tables and *.pepXML* summary. Finally, the raw files were converted with PEAKS Studio 10.5 and exported as *.mzXML*.

#### 2.8.2. LC-MS Data Integration and Filtration

A spectral library was built with Skyline [26,27,28] (v 20.1.0.155) using the *.pepXML* and *.mzXML* files without adding any additional FDR thresholds. MS raw files corresponding to affinity enriched samples and non-enriched aliquots were used for quantitation and a lock mass correction was applied during import. Extracted ion current (EIC) chromatograms were generated using the first 3 isotopes of each signal with a TOF resolving power of 20,000. Except for HSA peptides, all non-derivatized peptides were removed from the Skyline document to reduce the validation time. The retention time integration window was corrected when necessary. Isotopes indicating integration interferences were removed when possible, and likely chimeric spectra resulting from coeluting peptides displaying signals with a difference of less than 2 *m*/*z* units were annotated. Proposed ARP peptides were considered as confident when fulfilling the following criteria: (1) resolved chromatographic peak in the enriched fractions, (2) relative intensity in enriched fractions higher than in non-enriched fractions, (3) ARP reporter ions [16] present in the tandem mass spectra, (4) peptide sequence coverage above 50%, (5) fragment ions confirming the modification site, and (6) precursor error tolerance within ±2 standard deviations from the error mean (dataset-dependent, but typically between ±15 ppm). ARP peptides proposed with larger precursor errors or without fragments confirming the modification site were considered as ambiguous.

After all proposed peptides were manually confirmed, the Skyline ion mobility predictor function was used to automatically filter integration results in the ion mobility dimension [29] using a resolving power of 15. If necessary, the centers of ion mobility extraction windows were corrected manually. Integration results were exported as tables. Further data processing used scripts programmed in R (version 3.5.0).

#### 2.8.3. Enrichment Profile Estimation

The ARP-Plasma/ARP-OxHSA sample set was used to relatively quantify each peptide in the affinity-enriched fractions and the non-enriched aliquots. The loads of the enriched (5.8%) and non-enriched fractions corresponded to 11 µg (assuming no depletion) and 70 ng, respectively. The recovery percentage was calculated as

$$Recovery\;\%=100\;\times \;\frac{Area_{Enriched\;Fraction}}{Area_{Non-enriched\;Fraction}\times \frac{Load_{Enriched\;Fraction}}{Load_{Non-enriched\;Fraction}}}$$

It should be noted that the *Recovery* % calculation is a rough estimation that allows for visualizing the overall enrichment and depletion of peptides, assuming that the peptide quantities were always in the linear dynamic range and that matrix interferences were minimal.

## 3. Results

### 3.1. Derivatization, Digestion, and Enrichment Conditions

On the basis of the basic and acidic reaction conditions reported for ARP derivatizations, we evaluated three typical derivatization methods for human plasma. The derivatized plasma samples were separated by SDS-PAGE, blotted, and probed with extravidin-horseradish peroxidase to judge the ARP-derivatization degree on the basis of the quantitation of biotin (Figure S2A,B). Considering the numbers of detectable bands, we found that derivatization under neutral conditions yielded less and weaker bands than acidic conditions in the mass range below 60 kDa, whereas the presence of aniline as catalyst appeared to be the least efficient (Figure S2B, lanes 1–6). Interestingly, similar intensities were obtained for the HSA bands at ≈65 kDa for both acidic and neutral conditions at both ARP concentrations, while the addition of aniline again reduced the band intensity (Figure S2B,C). Independent of the pH, a reduction of the ARP concentration from 5 to 0.5 mmol/L reduced the HSA band intensities only by around 22% (acidic conditions) to 36% (neutral pH), indicating that the tenfold higher ARP concentrations likely allowed for a quantitative derivatization of reactive carbonyl groups. Derivatization of denatured plasma proteins in the presence of SDS (2%, *w*/*v*), likely to increase the accessibility of the carbonylation sites [20], did not further increase the intensities of immunoreactive bands (data not shown). Therefore, these reaction conditions were not further evaluated, especially as SDS was not quantitatively removed by subsequent ultrafiltration steps as reported by others [30]. All further experiments relied on acidic conditions (pH 2) and an ARP concentration of 5 mmol/L.

The excess of unreacted ARP and further uncharacterized contaminations detected after SPE with high signal intensities in ESI-MS after ARP derivatization at the peptide level and interfering with the peptide analytics [17] were efficiently removed here by ultrafiltration, reducing their signal intensities around 200-fold (Figure S3A,B). The reduced content of excess reagents is important, as ARP will compete with the derivatized peptides in affinity chromatography, reducing the enrichment efficiency for ARP peptides. Thus, protein derivatization was further evaluated due to the efficient removal of the reagent and small molecules in the samples up to a molecular weight of around 10 kDa, which also allowed for a favorable protein digestion in the ultrafiltration units (FASP) [24].

Another aspect considered was the bleeding of monomeric avidin from the affinity column due to the strong elution condition applied, which contaminated the reversed-phase (RP) column and thus interfered with LC-MS (Figure S4A-1,B-1). Ultrafiltration utilizing 10 kDa MWCO membranes removed monomeric avidin (≈16 kDa) mostly in the filtrate (Figure S4A-2,B-2), but it still accumulated slowly for consecutive injections or doubled sample loads on RP columns (see below). Membranes with lower cutoffs should be more efficient in removing avidin without reducing the recovery of peptides in the filtrate. Unfortunately, the tested ultrafiltration units (Microcon; Millipore) were not available with smaller pore sizes and alternative ultrafiltration units (5 kDa MWCO), which indeed should more efficiently remove monomeric avidin, but appeared not to be compatible with the applied conditions and LC-MS, as intense signals of contaminants suppressed the peptide signals.

**Figure 1:**
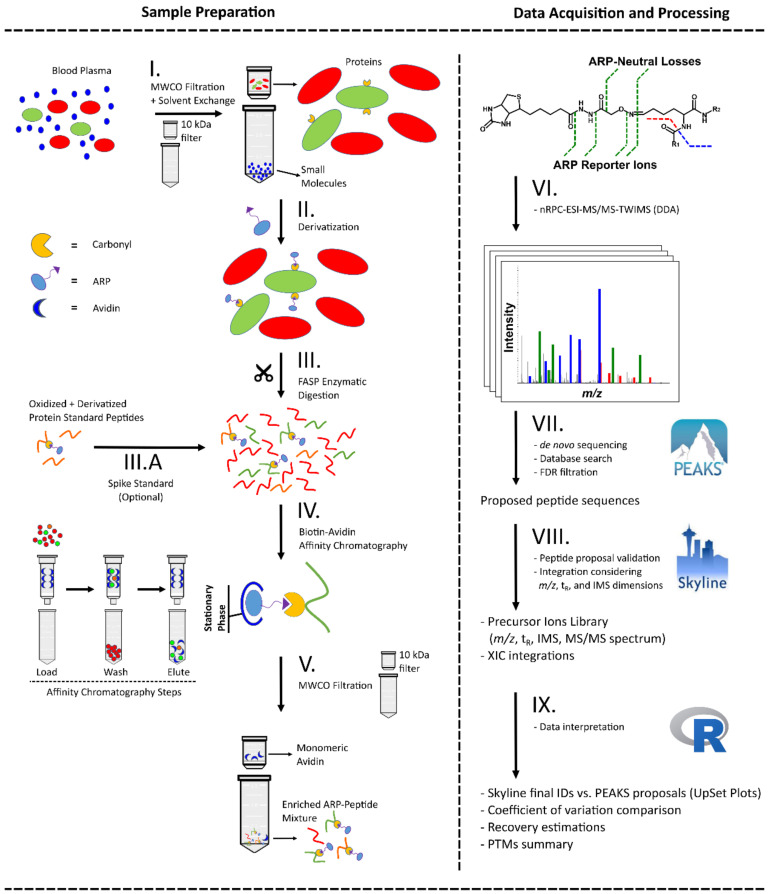
Schematic presentation of the analytical workflow applied to plasma samples for aldehyde reactive probe (ARP) peptide enrichment (**left**) and the following LC-MS)-based analysis from acquisition to data processing (**right**). Ultrafiltration of blood plasma was applied to simultaneously remove small molecules and reconstitute proteins in acidic conditions (**I**) to derivatize carbonylated proteins with ARP (**II**). Proteins were digested with trypsin using a filter-aided sample preparation (FASP) approach (**III**) and the resulting peptide mixture was split and either mixed with an ARP-labelled digest of a model protein (**III.A**) or directly enriched by avidin affinity chromatography (**IV**). The fractions were submitted to ultrafiltration to remove interfering monomeric avidin (**V**) and analyzed by nano reversed-phase chromatography coupled online to electrospray ionization tandem mass spectrometry with travelling wave ion mobility spectrometry (nRPC-ESI-MS/MS-TWIMS) in data-dependent acquisition (DDA) mode (**VI**). The generated tandem mass spectra were processed with a hybrid de novo and database search approach (**VII**) considering specific ARP fragmentation patterns. All proposed ARP peptides were validated by manual annotation of the mass spectra and considering both drift times in ion mobility spectrometry (IMS) and retention times in reversed-phase chromatography (RPC) (**VIII**). The filtered peptide list and corresponding peak areas were further processed (**IX**) to assess recovery, sample preparation workflow reproducibility, and protein carbonyl modification site location.

On the basis of these initial considerations, we applied a workflow consisting of six steps (Figure 1, left panel): (I) removal of small molecules from plasma and solvent exchange for *aq.* formic acid by ultrafiltration (10 kDa MWCO), (II) ARP derivatization of reactive carbonyl groups at the protein level in the ultrafiltration unit, (III) removal of ARP through ultrafiltration and FASP digestion, (IV) enrichment of ARP-labelled peptides in the filtrate by biotin-avidin affinity chromatography, (V) ultrafiltration of the eluted ARP peptides, and (VI) analysis of the filtrate using LC-MS/MS.

### 3.2. ARP Peptide Fragmentation Patterns

**Figure 2:**
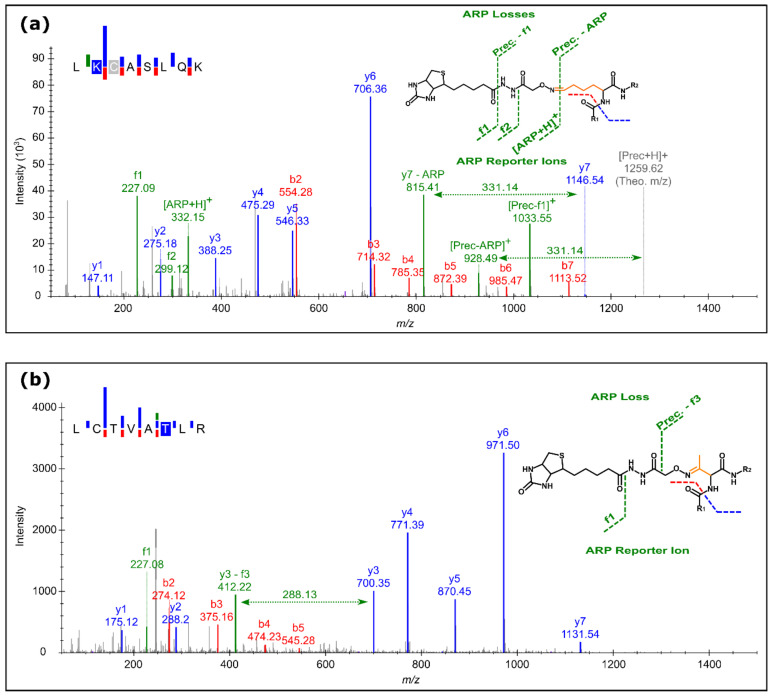
Fragmentation patterns of two doubly protonated precursors at *m*/*z* 630.315 (**a**) and *m*/*z* 622.816 (**b**) using collision-induced dissociation (CID). The indicated fragment ions confirmed ARP-derivatized tryptic human serum albumin (HSA) peptides, i.e., residues 197 to 204 with Lys199 modified as glutamic semialdehyde and residues 73 to 80 with Thr79 modified as 2-amino-3-ketobutyric. The small inserts indicate the proposed structures and fragmentation sites.

The tandem mass spectra of ARP peptides acquired in CID mode displayed several well-known reporter ions and modification-specific neutral or charged losses [13,16]. All spectra displayed a signal at *m*/*z* 227.085 (*z* = 1) corresponding to the biotin moiety of the ARP-tag, while the other reporter ions depended on the modification type. Oxidized Lys, Pro, and Arg residues (Table S1, Mods. 1, 8, 22) produced further intense signals at *m*/*z* 299.117 corresponding to a singly charged fragment ion of the ARP moiety and at *m*/*z* 332.139 representing the [ARP + H]^+^-ion (Figure 2a). Accordingly, precursor and y-ions carrying the PTM showed partial and full loss of the ARP-tag corresponding to 227.085 and 331.139 Da, respectively. The ARP-tag losses from precursor ion increased with the proximity of the PTM to the N-terminus. A different fragmentation pattern was observed for ARP-tagged oxidation products of Thr (Table S1, Mod. 6). The signal at *m*/*z* 227.085 was still intense, but no other previously encountered reporter ions were detected (Figure 2b). Instead, modified y-ions showed an additional modification-specific loss of 288.126 Da (Figure 2b). These general fragmentation rules were true for all OxHSA-derived tryptic peptides (Table S3, Figure S5) confirming their usefulness for a specific and confident identification of ARP peptides. Once confirmed for y-ions, these annotated losses were considered in the database search similar to the settings applied to the losses of phosphate and sulfate PTMs.

Even more importantly, the ARP reporter ions allowed for identifying tandem mass spectra presumably acquired for ARP-derivatized reactive carbonyls independent of the modification type and site. This, in combination with confident sequence tags provided by PEAKS, allowed for a reliable identification of ARP-tagged peptides including the following structures typically missed or misinterpreted: a serine oxidation product (+311.105 Da, Table S1, Mod. 7) generating the same fragmentation pattern as the aforementioned oxidation product of threonine, two alternative oxidation products of Lys (+298.074 Da, Table S1; Mod. 5) and Leu (+313.084 Da, Table S1, Mod. 10), a radical induced N-terminal backbone cleavage [6] on alanine and leucine residues (+312.089 Da, Table S1, Mod. 9), the metastable intra-cyclic deamidation intermediates of Gln and Asn residues (+314.105 Da, Table S1, Mod. 12), and assumed intra-cyclic dehydration products of Glu and Asp with structures identical to the Gln and Asn intermediates (+313.121 Da (Table S1, Mod. 11). The mass shifts corresponding to the new ARP MCO adducts were identified confidently only for derivatized, tryptic peptides of plasma samples spiked with ARP-OxHSA peptides.

Overall, ARP-specific signals allowed for the confident identification of PTMs initially not considered without impairing the identification of derivatized peptides at all. These signals were used at different stages of data processing: (1) identification of tandem mass spectra of likely ARP-tagged peptides using reporter ions in the low *m*/*z* range, (2) use of ARP-tag specific losses for an optimization of the search engine parameters, and (3) localization of the modification sites (Figure 1, right panel).

### 3.3. Enrichment Patterns and Matrix Interference Effects

**Figure 3:**
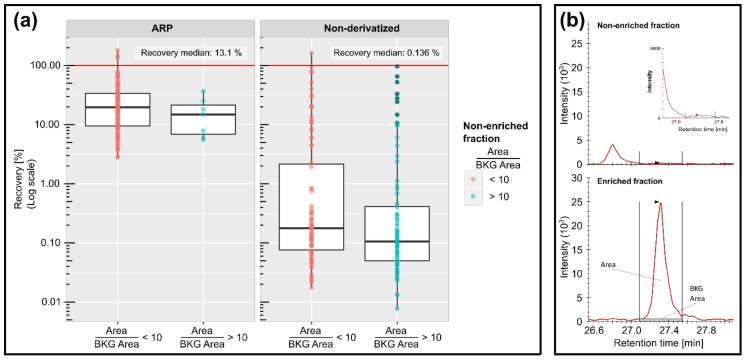
Box blots for the recovery rates of ARP and non-ARP-derivatized peptides (**a**) and extracted ion chromatograms (XICs) of the triply protonated ARP-labelled HSA peptide YIC[+57.022]ENQDSISSK[+312.089]LK at *m*/*z* 666.308 before (top) and after enrichment (bottom) by affinity chromatography (**b**). The recovery rates were grouped by area-to-background area (A/B) ratios in non-enriched fractions of below 10 (red) and above 10 (turquoise), presumably allowing a good quantitation.

ARP peptides and non-derivatized peptides were relatively quantified between the enriched and non-enriched fractions to judge the effectiveness of the enrichment and depletion process. In total, 108 confirmed ARP peptides were considered from a total of 491 peptides proposed by PEAKS for the LC-MS data of the affinity-enriched fraction of a plasma digest spiked with digested ARP-OxHSA (Table S3: “ARP-Plasma/ARP-OxHSA”). The recovery rates after affinity chromatography could only be judged for 94 peptides, as 37 peptides coeluted with isobaric substances present in the digest before affinity chromatography. However, only six proposed ARP peptides could be detected before enrichment in the EIC chromatograms with area to background (BKG) area ratios larger than 10, indicating recovery estimates from 6 to 37% (Figure 3, ARP, blue). These recovery estimations were further challenged by the different matrix composition before and after enrichment, but the comparison of the enrichment patterns appeared to be valid. Many non-derivatized peptides were still present in the enriched fractions, but at significantly depleted levels with only a few HSA peptides detected with recovery rates similar to the ARP peptides. Astonishingly, some non-derivatized HSA peptides identified in the enriched fractions were not detected prior to enrichment (Figure 3, non-derivatized, red), similar to most ARP peptides, indicating that they were significantly enriched. Most of these peptides eluted late from the RP column, suggesting that hydrophobic peptides were unspecifically enriched. Additionally, 10 HSA-derived peptides showed recovery rates from 5 to 165% contained an “HPY” sequence motif, which reportedly binds with high affinity to avidin [31]. Regardless of the presence of non-derivatized peptides, the specific enrichment of ARP peptides was apparent from the 100-fold difference between the medians of the recovery percentages of ARP peptides and non-derivatized peptides (Figure 3a). Moreover, the peak areas of 90% of ARP peptides and 58% of non-derivatized peptides showed coefficients of variation (CV) below 20% (Figure S6), confirming the reproducible trapping of ARP peptides.

### 3.4. Increase in Sensitivity of ARP Peptides with Sample Preparation Upscale

Only 21 of the 131 ARP peptides identified in the same plasma sample spiked with ARP-OxHSA were detected in ARP-Plasma, which reflects the low basal level of reactive carbonyl groups in healthy individuals. More modified peptides might be detected by using larger protein amounts, although this would be limited by the interference from non-derivatized peptides. Therefore, controlled dilution series were prepared by diluting identical aliquots of one ARP-OxHSA FASP digest in increasing volumes of non-derivatized plasma digests. This plasma was digested in solution to avoid protein precipitation issues during ultrafiltration of FASP [17]. This increased the contents of partially digested peptides (up to 10 kDa) eluting late in the gradient, but it was still possible to assess matrix effects on the signal intensities of the targeted ARP peptides. More limiting were the increased signal intensities of other matrix signals that often triggered a tandem mass spectrum reducing the number of identified ARP peptides among replicates (Figure S7A). Furthermore, quantitation was less reproducible for higher plasma ratios (Figure S6B) due to ionization suppression or coeluting isobaric plasma compounds fragmented together with the targeted peptide. Despite these limitations, the signal response of all 153 ARP-peptides (Table S3: “ARP-OxHSA Dilution Series”) was mostly independent of the dilution factor. Relative to the average peak areas of the ARP peptides obtained for the 1:9 mix, the peak area average of the corresponding peptides in the 1:49 and 1:249 mixes showed median ratios of 1.05 and 0.95, respectively (Figure S6C). This clearly indicates an efficient trapping of the targeted analytes even at 25-fold higher plasma concentrations, allowing for an upscaling from 0.2 to 5 mg plasma proteins. However, the following studies relied on 2 mg plasma proteins to reduce the background on LC-MS and to prevent memory effects seen especially for some hydrophobic substances after multiple sample injections.

**Figure 4:**
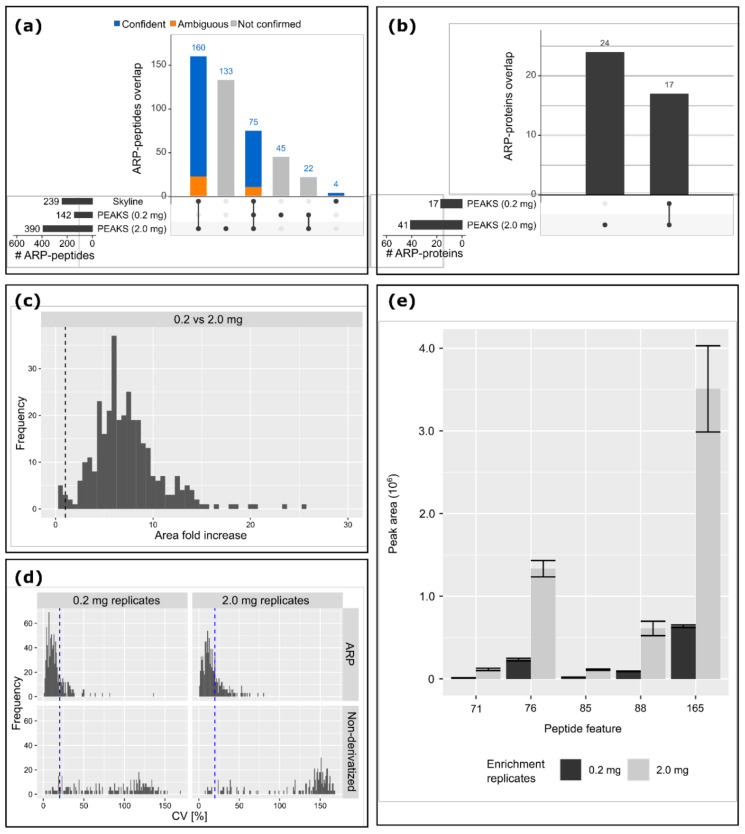
Identification and quantitation of ARP peptides in tryptic plasma digests starting with low and high protein quantities of 0.2 mg and 2 mg plasma protein, respectively. (**a**) UpSet plot of ARP peptides proposed by PEAKS (5% false discovery rate (FDR), peptide level) and after validation by Skyline. The total number of peptides per classification are displayed in horizontal bars and overlapping identifications in vertical bars. The confidence level is indicated by color, i.e., blue, orange, and gray indicating confident, ambiguous, and unconfirmed sequences, respectively. (**b**) UpSet plot of proteins confidently identified by Skyline on the basis of all confidently validated ARP peptides. (**c**) Increase of peak areas obtained for confidently identified ARP peptides when upscaling the analytics from low to high plasma protein quantities. (**d**) Coefficients of variation (CV) for ARP peptides (top) and non-derivatized peptides (bottom). (**e**) Peak areas of HSA peptide LKC[+57.021]ASLQK modified on the second residue (Lys199 in HSA) by mass shifts of +475.174 Da (71), +355.131 Da (76), +385.142 Da (85), +369.147 Da (88), and +312.089 Da (165).

After validation and integration within Skyline, the peptide list was compared to the 435 peptides suggested by PEAKS (Figure 4a). A total of 239 ARP peptides were confirmed, 235 in the replicates of the 2.0 mg sample and 75 in the 0.2 mg replicates, corresponding to 41 proteins and 24 proteins, respectively (Figure 4a,b, Table S3: “ARP-Plasma Upscale”). Manual revision of the tandem mass spectra revealed four false ARP peptide proposals and excluded many others due to incorrect assignment of the number of PTMs per peptide by the search engine; for example, peptide LKEC[+369.147]C[+57.022]EKPLLEK (HSA 275-286; numbers corresponds to the mature sequence of HSA-isoform P02768-1) proposed by PEAKS with two PTMs at Cys278 (ARP acrolein Michael adduct, Table S1, Mod 29) and Cys279 (carbamidomethylation). However, manual interpretation implied three modifications, i.e., both cysteine residues were carbamidomethylated (y_9_ ion indicating Cys278[+57.022]) and oxidized Lys276 derivatized by ARP, representing the remaining mass shift of 312.125 Da, which was deduced from the b_2_- and y_11_-331.1-signals (Table S1, Mod 8). The deduced sequence was LK[+312.089]EC[+57.022]C[+57.022]EKPLLEK (identifiers for the MS/MS spectra provided in Table S4). Among the 196 proposed but unconfirmed ARP peptides were many supposedly carrying two or more ARP labels, which showed a poor spectral quality and poor sequence coverage. However, peptides with multiple carbamidomethylated Cys residues were confidently identified. As all confirmed Cys-containing peptides, except one, were alkylated, and the datasets were processed again using carbamidomethylation as a fixed Cys modification. During the PTM assignments, PEAKS DB and PEAKS PTM can reconsider PSMs with -10lgP confidence scores lower than 30 with different PTMs than those assigned as fixed, such as cysteine carbamidomethylation, allowing it to identify HSA peptide with Cys34[+369.147] (Table S1, Mod 29).

Enriching tenfold higher protein quantities improved the signal-to-noise ratios with 98.2% of the confirmed ARP peptides displaying on average 7.3-fold higher signal intensities (Figure 4c). Quantitation reproducibility of ARP peptides was 76% and 68% in 2.0 and 0.2 mg replicates, respectively, with CVs lower than 20% (Figure 4d). Previously, a decrease in quantitation reproducibility was observed with increasing matrix contents, but the increased abundance of ARP peptides compensated some matrix effects.

**Figure 5:**
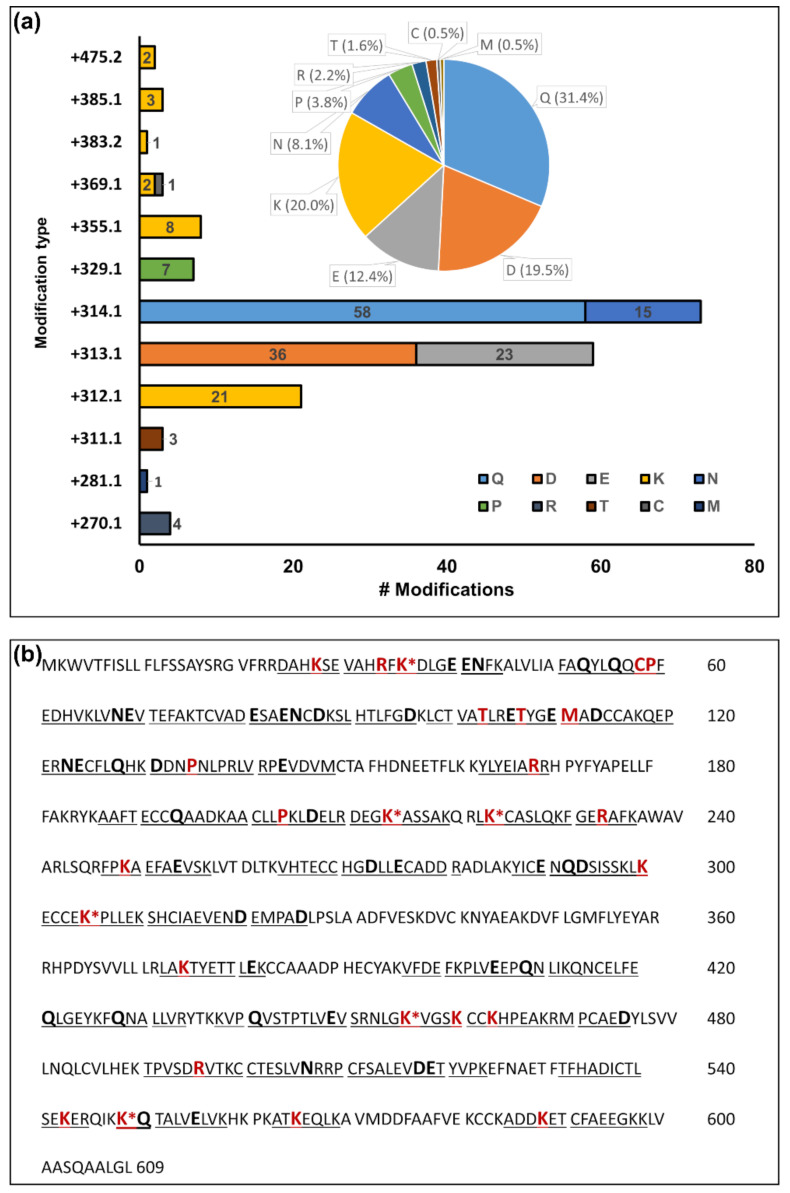
Frequency of modification types and modified amino acids (**a**) and respective modification sites (**b**) identified in HSA using plasma with a total protein content of 2 mg. The bar plot represents the total number of unique residues modified by each modification type and the pie diagram the proportion of each modified amino acid. The asterisk (*) indicates residues where more than one modification was confirmed. Bold capitals highlight modifications at Gln, Asn, Glu, and Asp. Bold red capitals highlight all other residues modified by reactive carbonyls.

The higher signal intensities also improved the signal-to-noise ratio of the tandem mass spectra, allowing the confident mapping of 215 carbonyl groups at 192 sites and 35 more ambiguous modifications requiring a confirmation by targeted analysis (Figure 5a, Table S3: “ARP-Plasma Upscale”). Modifications corresponding to 12 different mass shifts were detected at 10 amino acids (Figure 5a).

The most common modification was, to the best of our knowledge, a previously unreported mass shift of +314.105 Da observed at 58 Gln and 18 Asn residues in 19 proteins. Prior to derivatization, the corresponding carbonyl modification would have an exact mass shift of +0.984 Da, which is annotated in unimod (https://www.unimod.org) as Gln/Asn deamidation. As we did not find any reports on an ARP reactivity towards carboxyl groups or amides, a likely explanation is that the acidic conditions capture cyclic imide intermediates of Gln/Asn [32], which can react with ARP as reported for some cyclic imides [10]. In this respect, the observed mass shift of +314.105 Da might correspond to an ARP oxime formation with a cyclic intermediate that induces a ring opening instead of a water loss (Table S1, Mod. 12). A similar structure was considered for Glu and Asp with an exact mass shift of +313.121 Da (Table S1., Mod. 11). This mass shift was detected in 95% of all cases on C-terminal Asp residues suggesting a peptide backbone cleavage via cyclic isoimide intermediates [33] or Asp-specific proteases [34]. An in vitro experiment was performed with a mixture of synthetic peptides containing Gln/Asn and Glu/Asp to ensure that these modifications were not artifacts of the derivatization conditions applied here. Interestingly, no significant degradation was observed after 24 h, at least without enrichment, neither in acidic (1% formic acid (*v*/*v*)) nor near neutral pH (25 mmol/L ammonium bicarbonate) (Figure S8).

**Figure 6:**
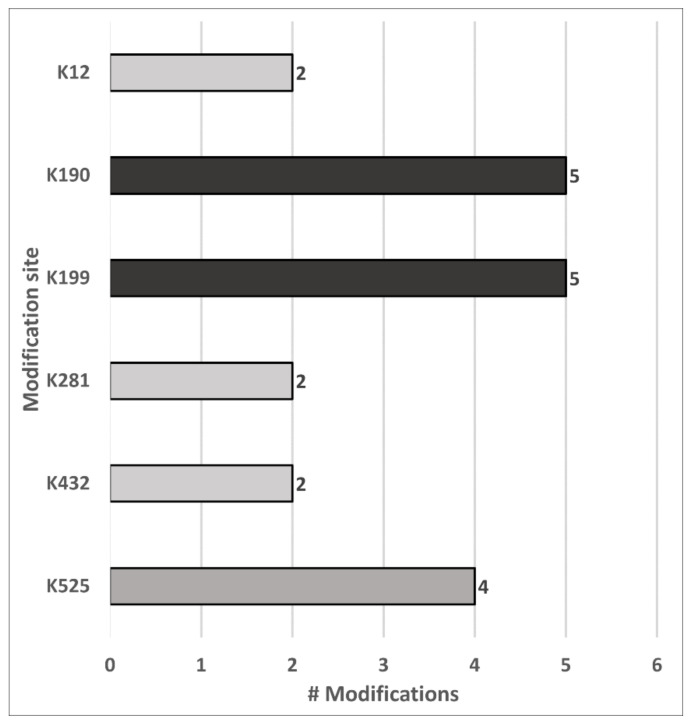
Lysine residues in HSA identified with different ARP-labelled modification types.

Despite the speculative structures and mechanisms, the location of the mass shifts was confident, and their consideration allowed a better overall confidence of annotation by eliminating false positives. The next common mass shift of +312.089 Da was observed at 21 sites in seven proteins, mainly at Lys residues (Table S1, Mod. 8). Lys residues were also observed with mass shifts of +475.174 Da indicating two glycation sites, +385.142 Da indicating three malondialdehyde or methyglyoxal Michael adducts, +383.163 Da indicating one crotonoaldehyde Michael adduct, +369.147 Da indicating an acrolein Michael adduct, and +355.131 Da indicating eight aldoamine products (Table S1, Mods. 25, 29, 32, 33/34, 48). Other modifications corresponding to derivatized adducts of MCO products (Table S1, Mods. 1, 2, 6, 22) were located on seven Pro residues (+329.116 Da), four Arg residues (+270.067 Da), three Thr residues (+311.105 Da), and one Met residue (+281.111 Da). Lastly, a shift of +369.147 Da observed on Cys34 in HSA indicated an acrolein adduct. Most modifications (87) and modification types were identified in HSA, with 73 of the 171 modification sites mapped. Interestingly, six Lys residues were modified in HSA by more than one type of modification (Figure 6).

## 4. Discussion

The detection of reactive protein carbonyls in patients with chronic renal failure [35], Alzheimer’s disease [36], and several other diseases related to oxidative distress [37,38] at elevated levels has triggered further research to study their potential as diagnostic and prognostic biomarkers [8]. However, most methods quantify only the total content of reactive carbonyl groups in a sample [7] and thus do not provide any information how individual carbonylation sites might relate to the disease specific pathology [39]. Recent research has focused more on identifying protein carbonylation sites in easily accessible tissues or body fluids, such as blood plasma [17,20]. This has provided important information about distinct modification sites in many proteins, but reproducible quantitative data for these potential biomarkers are mostly missing. Thus, we intended to develop a robust protocol to identify protein carbonylation sites by untargeted LC-MS and to reproducibly quantitate these carbonylation sites at the peptide level using targeted LC-MS.

### 4.1. ARP-Specific Fragmentation Patterns Improved Identification Accuracy

Since SEQUEST was introduced in 1994 [40], multiple algorithms have been developed to improve the processing speed and accuracy of peptide identifications from bottom-up proteomics studies. Most tools rely mainly on characteristic peptide backbone cleavages generated, for example, after collision induced dissociation (CID), electron capture dissociation (ECD), and electron transfer dissociation (ETD), allowing a reliable identification of unmodified peptides. These tools allow also for searching for posttranslational modifications, but typically only by their increment mass, ignoring the unassigned intense signals produced from fragmentations of the modified side chains, which often results in low confidence scores. These weaknesses have been solved for common PTMs, such as phosphorylated Ser, Thr, and Tyr residues prone to neutral losses of HPO_3_ or H_3_PO_4_, but remain a major problem for rarely studied enzymatic and especially non-enzymatic modifications. Most publications analyzing derivatized carbonylation sites at the peptide level relied on MASCOT [3,11,12,13,14,15,16,18,19,20,21,22,41,42,43,44], which is very reliable in protein identification. However, the scoring of derivatized peptides is negatively biased compared to unmodified peptides even when tag-specific neutral losses are considered or the mass spectra are cleared from signals corresponding to tag-specific fragment ions to improve the identification scores of derivatized peptides [16,20]. The de novo sequencing approach implemented in PEAKS is not impaired by additional signals that result from side chain or tag fragmentations. Moreover, by limiting its search space to proposed de novo tags, it is able to consider hundreds of possible PTMs in a single database search, simplifying the consideration of many PTMs. The current study relied on 48 distinct derivatized carbonylation adducts, with 15 being confidently identified in human plasma proteins. Importantly, the software allowed for the discovery of carbonyl PTMs originally not considered by using sequence tags identified by the software that were matched to a protein sequence in the database, but were interrupted by only one residue with a mass shift not fitting to the considered PTMs. After localizing the mass shifts to a specific residue and in combination with the use of ARP-specific reporter ions, we were able to map ten previously missed ARP-carbonyl adducts.

### 4.2. Protein Derivatization Improved the Enrichment Efficiency for Target Analytes

The removal of non-target biotinylated species prior to avidin affinity chromatography is essential to increase the sensitivity and to decrease the complexity of affinity-enriched samples while keeping the sample volumes as low as possible to obtain high peptide concentrations in the eluate. Unfortunately, the simple and efficient removal of reagents by ultracentrifugation is not possible at the tryptic peptide level [17]. Theoretically, differences in hydrophobicity might allow for a separation by RPC, but ARP and biotinylated contaminants eluted in the same retention time window as many derivatized tryptic peptides (data not shown). Similarly, the mixed hydrophilic-hydrophobic HLB phase used in a previous study [17] did not efficiently remove the excess of reagent and biotinylated contaminants, at least for the conditions tested here. Additionally, the hydrophobic contaminants competed with the peptides on the RP column, limiting the sample loads and thus the overall sensitivity. ARP derivatization of peptides is most likely more efficient than for proteins, as carbonyl sites can be buried inside of proteins. Furthermore, the tryptic cleavage might be more efficient, as the bulky ARP tags may mask nearby cleavage sites. However, in our hands, the derivatization at the protein level appeared more viable due to the removal of contaminants.

### 4.3. Unexpected Reactive Carbonyl PTMs

The initial study relied on reactive carbonyl groups reported for MCO, LPP, and AGE products. However, the confidently identified mass shifts of +314.105 Da at 73 Gln/Asn- and of +313.121 Da at 59 Glu/Asp-residues suggested further reactive groups to be considered. The mass shifts correspond most likely to ARP adducts of cyclic imide/isoimide intermediates (Table S1, Mods 11 and 12) that can be generated at physiological conditions [32] and isolated at acidic pH, as applied here for derivatization, i.e., 1% formic acid. Notably, the mass shift obtained for Asp was highly specific for C-terminal Asp residues, which may indicate a succinimide-dependent protein cleavage [45] or less likely a caspase-related cell suicide mechanism [34] or cleavage during tryptic digestion for example by blood proteases.

The pathways generating the proposed cyclic imides and isoimides were beyond the scope of this study, but they might be relevant for studies on protein deamidation and caspases. Nevertheless, they should be considered as a possible pitfall when quantifying total carbonyl contents. The dispersion across all proposed proteins and the relative intensities of the EIC chromatograms of peptides derivatized with these Gln/Asn and Glu/Asp modifications suggested rather high contents that may partially explain the observed differences in the Western blots. Presumably, other derivatization reagents, such as 2,4-dinitrophenylhydrazine (DNPH) [7,46], will also react with these PTMs. Interestingly, cysteine residues oxidized to sulfenic acid [47] are reactive towards DNPH in acidic conditions, further illustrating the ambiguity of total carbonyl quantitation methods, although this Cys modification was not observed in the current study. This implies a need of specific LC-MS-based methods that can distinguish different carbonyl groups derivatized with hydrazine and hydroxylamine-based tags. The observed cross-reactivity can be reduced by using neutral derivatization conditions [15,20], but not prevented as the deamidation half-time of Gln/Asn has been shown to be sequence-dependent, ranging from 0.5 to 20,000 days at physiologically relevant conditions [32].

Disregarding these dominant PTMs might lead to false-positive peptide identifications. For example, the mass shift resulting from an ARP glyoxal adduct on cysteine (+C_14_H_21_N_5_O_5_S, Table S1, Mod 30) were identical to the combined mass shifts of carbamidomethylation (+C_2_H_3_NO) on cysteine and a Gln/Asn imide ARP adduct (+C_12_H_18_N_4_O_4_S, Table S1, Mod 12). Once the Gln/Asn imide ARP adduct was considered, we were able to correct ambiguous peptide sequences initially proposed with an ARP glyoxal PTM.

### 4.4. Carbonylation Site Specificity

The established protocol allowed identifying multiple types of carbonyl modifications and, more importantly, mapping them to specific sites. Among them, the carbonyl-groups of 55 ARP peptides originated from MCO, AGE, and LPP products. The majority of these peptides were mapped to HSA with 26 direct amino acid oxidation products and 17 reactive carbonyl species (RCS) adducts. This significant reactivity towards reactive electrophiles is well known [48,49,50,51], and HSA was proposed as an important scavenger of RCS in plasma [50]. Interestingly, 83% of the RCS adducts were located on one of six lysine residues of HSA, with Lys190, Lys199, and Lys525 matching previously reported preferential targets of 4-hydroxy-2-*trans*-2-nonenal (HNE) in vitro [50]. Although, no HNE modifications were confidently identified in the current study, the overlap of these reactive sites supports the specific reactivity towards RCS and the importance of mapping the modification sites. Despite the high number of adducts reported for Cys34 [52], this study confidently identified only one acrolein adduct at this site. This apparent contradiction illustrates the benefits and limitations of the workflow established here, as all adducts reported by Grigoryan et al. [52], for example, contained adducts of low molecular weight species lacking reactive carbonyls. However, they also showed that most disulfide adducts were lost when the proteins were digested after reduction with TCEP. Intriguingly, the acrolein adduct on Cys34 identified here was not reported by these authors, indicating that low abundant reactive carbonyls can only be identified after peptide enrichment. Curiously, no HNE adducts could be confirmed in the current dataset, although it is considered as one of the most abundant LPP products in vivo. A similar observation was reported for an in vivo study on rat mitochondria [11], identifying 49 distinct RCS Michael adducts at 39 sites in 27 proteins sites, but not a single HNE adduct. The authors proposed a lack of reactivity of ARP towards the predominantly hemiacetal conformation of HNE Michael adducts [53] as a likely reason for missing the expected HNE adducts. However, in a follow-up study, the authors could identify HNE adducts after ARP derivatization when rat mitochondria were exposed to increasing levels of HNE in vitro [15]. Thus, it is more likely a matter of the limit of detection that prevents detection of HNE adducts in vivo.

Besides the modification types mentioned above, two of the five commonly modified Lys residues of HSA were also glycated, which corresponds to reports indicating that Lys199 and Lys525 are major glycation sites in vivo [49,54,55]. The remaining 12 confidently identified ARP peptides (Table S3: “ARP-Plasma Upscale”) corresponded to direct amino acid oxidation products from seven Lys, three Pro, and one Thr residues mapped to immunoglobulin heavy constant gamma 1, fibrinogen alpha chain, fibrinogen beta chain, histidine-rich glycoprotein, immunoglobulin lambda-1 light chain, immunoglobulin lambda constant 2, and transmembrane protein 198. It should be noted that many of the peptides mapped to the immunoglobulins were not unique but corresponded to immunoglobulin protein groups (Table S3: “ARP-Plasma Upscale”). The large homology of immunoglobulins should be considered, as it may explain the large number of carbonylated immunoglobulins observed in breast cancer patients [22].

### 4.5. Peptide Level Enrichment Improved the Identification Accuracy of Carbonylated Proteins

In this study, a peptide-level enrichment approach was applied due to the increased likelihood of locating protein carbonylation sites [15], but important implications for accurate identifications of carbonylated proteins were observed for both peptide and protein enrichment approaches. The unspecific enrichment of non-derivatized peptides by avidin affinity chromatography has to be considered for the final list of proposed carbonylated proteins, because they may lead to the false identification of carbonylated proteins. Thus, carbonylated proteins should not be proposed on the basis of the detection of peptides in enriched fractions but only if confirmed derivatized peptides were observed. In our hands, even extensive washing (≈50 column volumes) did not remove all non-derivatized peptides due to their strong “unspecific” binding to avidin or specific enrichment via sequence motifs with high binding affinity to avidin. On the basis of these stringent considerations, we found that only a modest number of 43 carbonylated proteins in human plasma was considered, despite 79 proteins being proposed by PEAKS from all tandem MS spectra in the enriched fractions.

## 5. Conclusions

This study addressed several aspects of sample preparation that are important in our mind and have not been thoroughly investigated in previous bottom-up proteomics studies targeting reactive carbonylation sites to the best of our knowledge. The current study indicates the benefits of focusing on ARP-specific ions in targeting reactive carbonyl groups in proteins despite reports indicating their supposedly detrimental influence in peptide identifications. Finally, the simultaneous optimization of sample preparation and data processing provided a workflow that can both identify many of reactive carbonyl PTMs and reproducibly quantitate the corresponding peptides. Although, the current study did not address the biological relevance of the identified carbonylated proteins and individual carbonylation sites, the baseline detection of derivatized carbonylated peptides from a healthy individual will allow for relative quantitative studies between healthy and diseased cohorts in the context of oxidative stress conditions.

## Data Availability

The mass spectrometry proteomics data and R scripts have been deposited to the ProteomeXchange Consortium via the PRIDE [56] partner repository with the dataset identifier PXD023738 and 10.6019/PXD023738. Additionally, all MS/MS spectra of confirmed ARP peptides can be accessed using their universal spectra identifiers (www.psidev.info/usi; accessed on 10 December 2020) provided in Table S4, Supplementary File 2.

## Supplementary Materials

The following are available online at https://www.mdpi.com/2076-3921/10/3/369/s1. Figure S1: Sample preparation schemes. Figure S2: Evaluation of ARP derivatization conditions. Figure S3: Interference in protein and peptide derivatization. Figure S4: Ultrafiltration to remove avidin. Figure S5: HSA sequence coverage for plasma spiked with ARP-OxHSA. Figure S6: Integration reproducibility of peptides in enriched fractions. Figure S7: Identification and quantitation reproducibility summary plots from ARP-OxHSA dilution series dataset. Figure S8: Stability of Gln/Asn- and Glu/Asp-containing peptides in acidic conditions. The following are available online at https://www.mdpi.com/2076-3921/10/3/369/s2: Table S1: Mass shifts and structures of all derivatized carbonyl modifications considered in this study. Table S2: Database search FDR thresholds. Table S3: List of confirmed ARP peptides. Table S4: Universal spectrum identifiers (USIs).
