# Supplementary material for: A Workflow towards the Reproducible Identification and Quantitation of Protein Carbonylation Sites in Human Plasma
Source: Antioxidants (Basel). 2021 Mar 1;10(3):369. doi: 10.3390/antiox10030369 (PMC7999155; doi:10.3390/antiox10030369)
Supplement: Supplementary file 1 [file antioxidants-10-00369-s001.pdf]

Protein Carbonylation Sites in Human Plasma: A Workflow towards Reproducible Identification and Quantitation of Protein Carbonylation Sites

Juan Camilo Rojas Echeverri[1,2], Sanja Milkovska-Stamenova[1,2] and Ralf Hoffmann[1,2,*]

[1] Institute of Bioanalytical Chemistry, Faculty of Chemistry and Mineralogy, Universität Leipzig, Leipzig, Germany
[2] Center for Biotechnology and Biomedicine, Universität Leipzig, Leipzig, Germany
* Correspondence: bioanaly@rz.uni-leipzig.de

Supporting information

Figure S1: Sample preparation schemes.

Figure S2: Evaluation of ARP-derivatization conditions.

Figure S3: Interference in protein and peptide derivatization.

Figure S4: Ultrafiltration to remove avidin.

Figure S5: HSA sequence coverage for plasma spiked with ARP-OxHSA.

Figure S6: Integration reproducibility of peptides in enriched fractions.

Figure S7: Identification and quantitation reproducibility summary plots from ARP-OxHSA dilution series data set.

Figure S8: Stability of Gln/Glu- and Asn/Asp-containing peptides in acidic conditions.

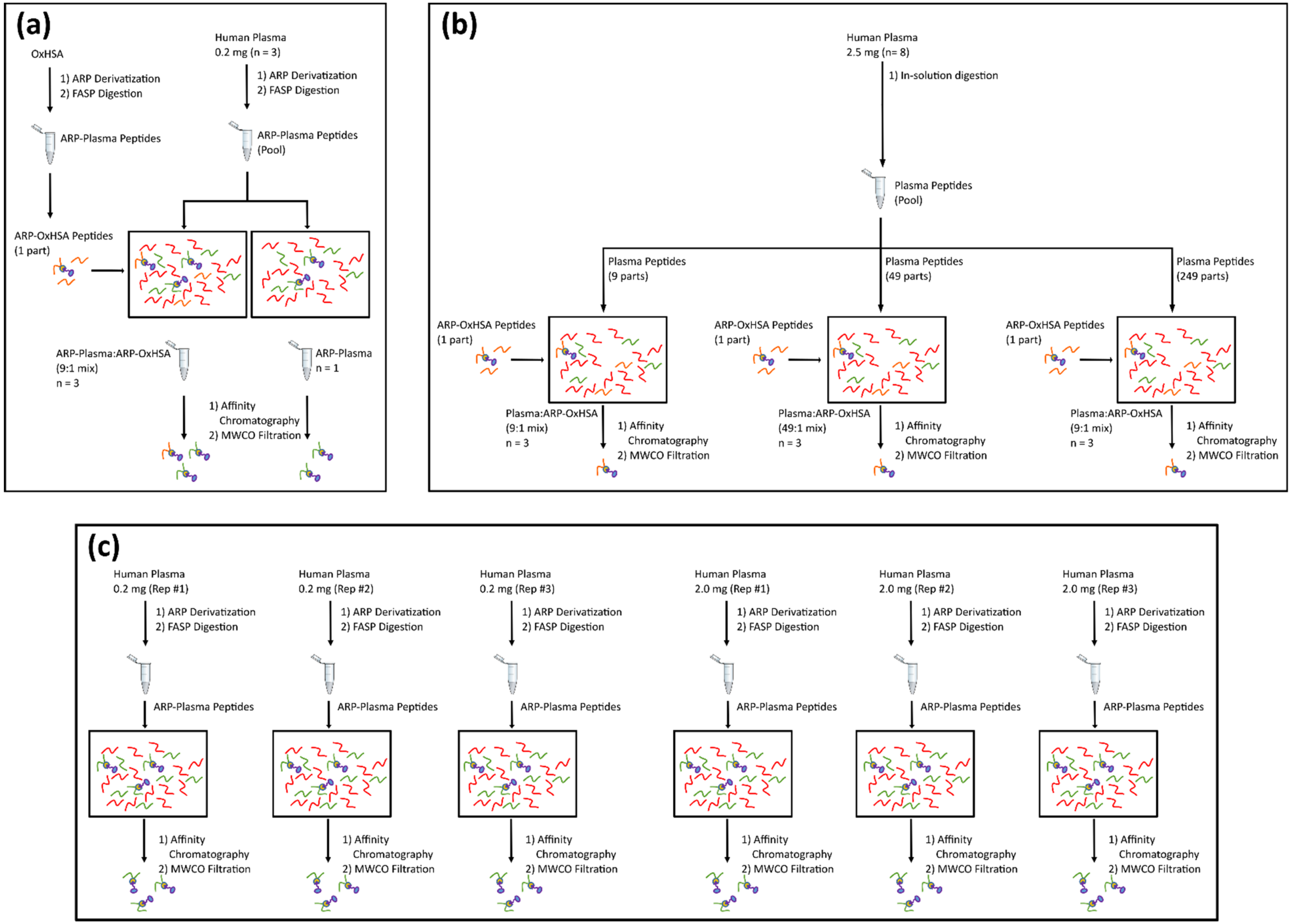

Figure S1. Experimental setup used to evaluate the enrichment reproducibility (ARP-Plasma:ARP-OxHSA data set; panel (**a**), the matrix interference effects (Plasma:ARP-OxHSA data set; panel (**b**), and the reproducibility of the whole procedure (ARP-Plasma Upscale data set; panel (**c**).

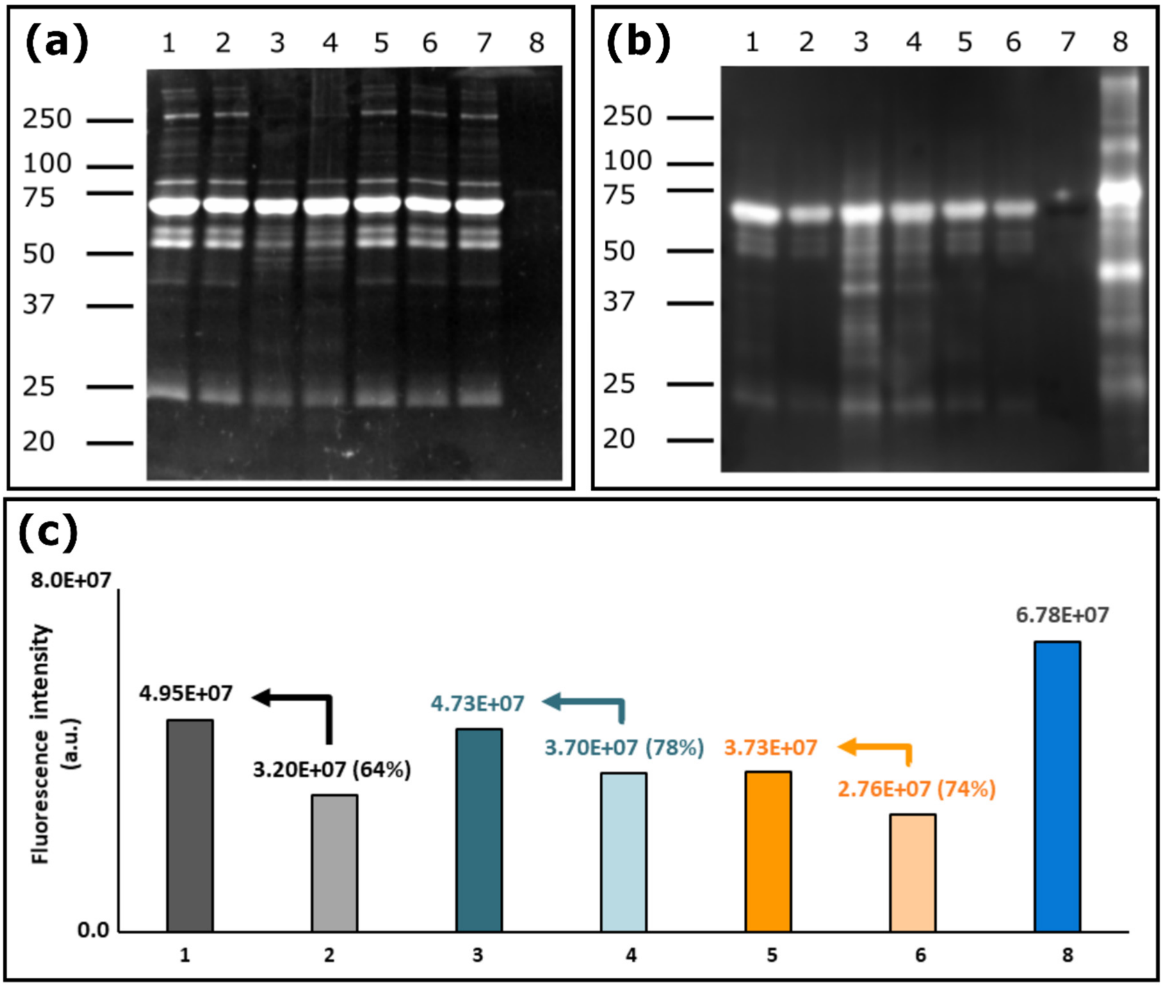

Figure S2. SDS-PAGE (**a**), corresponding anti-biotin Western blot (**b**), and fluorescence intensities measured for the HSA bands (~65 kDa) of each lane on the membrane (**c**). The gel was stained after SDS-PAGE with Oriole™ fluorescent stain and subsequent colloidal Coomassie Brilliant Blue G-250. After semidry-blotting onto a PVDF-membrane, the membrane was probed with extravidin coupled to horseradish peroxidase and incubated with Immuno Blue Western Blotting. Human plasma samples (1 µg protein) loaded on the gel were incubated with ARP at concentrations of 5 mmol/L (odd lane numbers) or 0.5 mmol/L (even lane numbers) overnight in ammonium bicarbonate (25 mmol/L, lanes 1 and 2), aqueous formic acid (1 % (v/v), lanes 3 and 4), and aniline (0.1 mol/L) in ammonium bicarbonate (25 mmol/L, lanes 5 and 6). Human plasma incubated in parallel in ammonium bicarbonate (25 mmol/L) without ARP (lane 7) was added as a negative control and biotinylated bovine serum albumin (20 ng) was added as a positive control (lane 8).

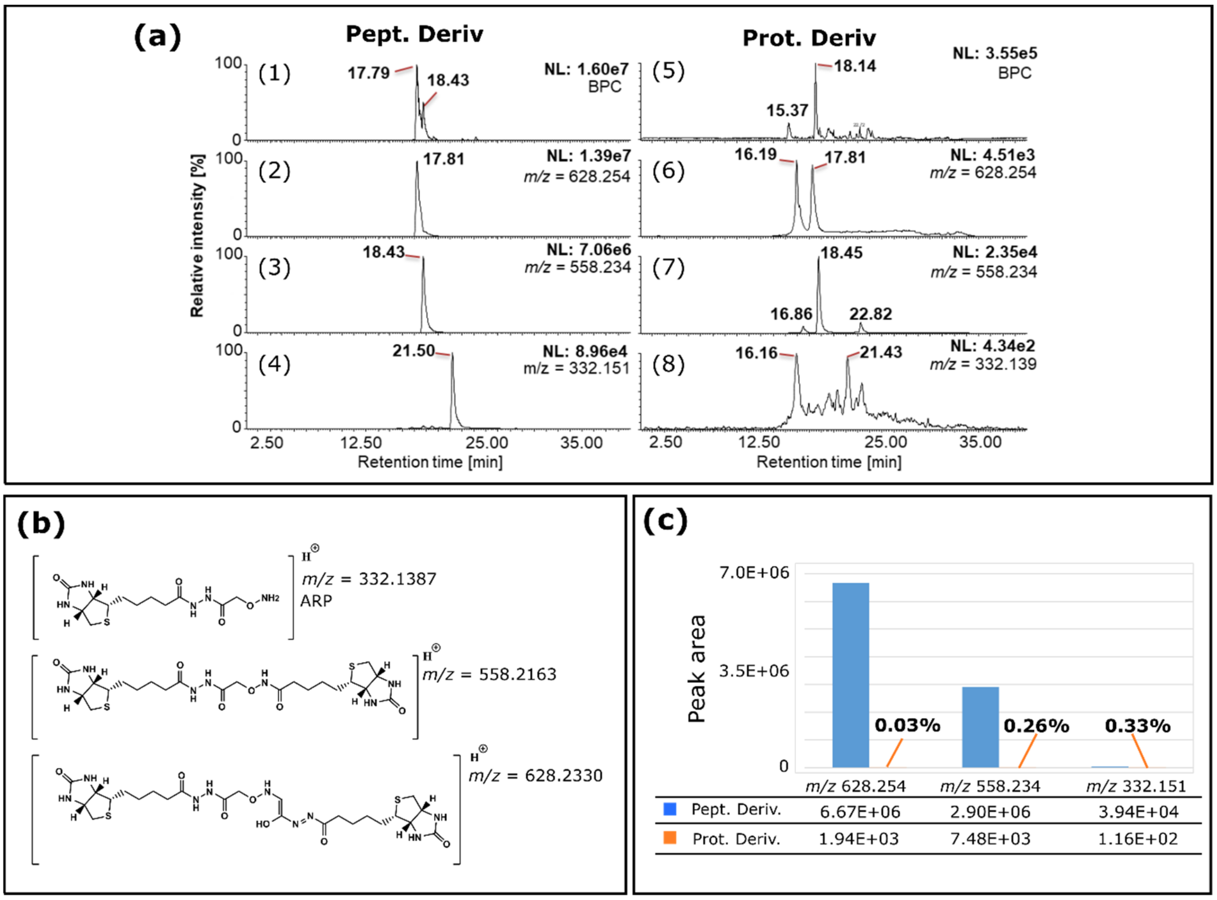

Figure S3. Base peak and extracted ion chromatograms (**a**) of enriched samples obtained after peptide (left) or protein derivatization (right). Structures of ARP and proposed biotinylated contaminants with respective *m*/*z*-values of the singly protonated species (**b**). Peak areas of biotinylated species obtained after peptide and protein derivatization (**c**).

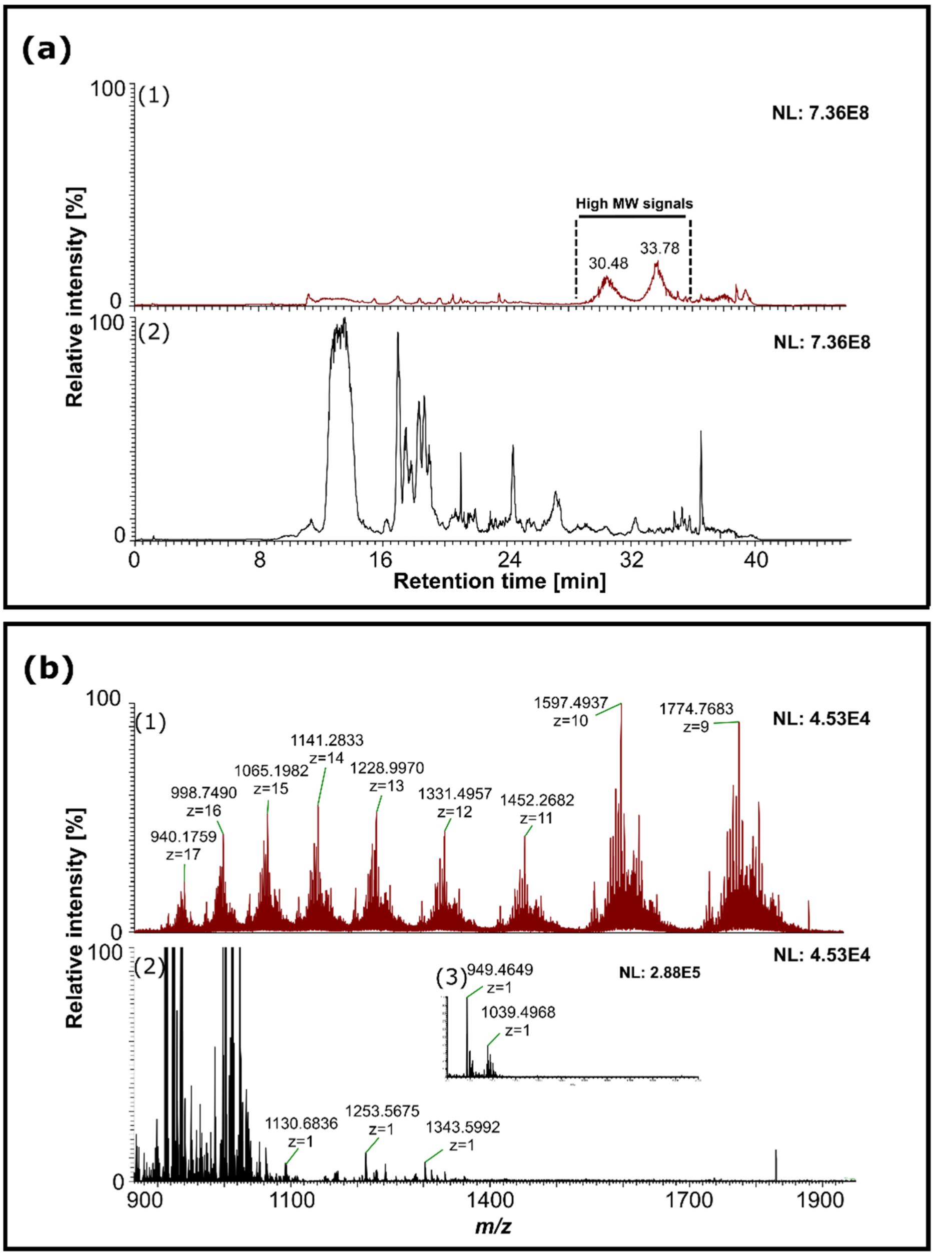

Figure S4. Total ion current chromatograms of sample preparation blank without filtration (top) and enriched peptide fraction after ultrafiltration (10 kDa MWCO, bottom) (**a**). Average mass spectrum from *m*/*z* 900 to *m*/*z* 2000 for compound eluting between 29.3 and 31.8 min from the sample preparation blank (1) and the enriched peptide fraction (2 & 3) (**b**).

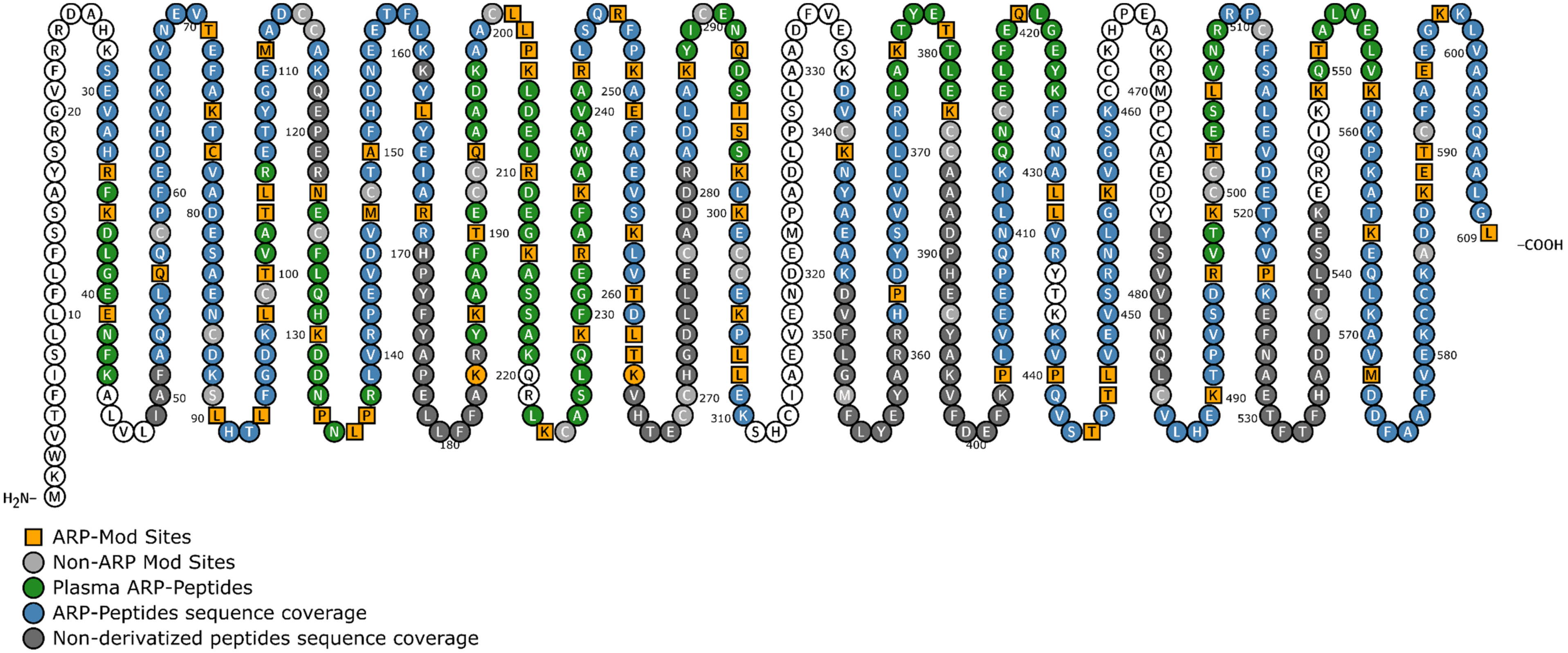

Figure S5. Human serum albumin (HSA) sequence coverage obtained for the ARP-Plasma:ARP-OxHSA data set. Sequence coverage from non-derivatized peptides, ARP-peptides detected in ARP-OxHSA-containing samples, and ARP-peptides detected in plasma are displayed with dark grey, blue, and green circles, respectively. Yellow squares indicate confident ARP modification sites. Other modifications, such as carbamidomethylation, methionine oxidation, and carbamylation, are represented by light grey circles. Image generated with PROTTER.

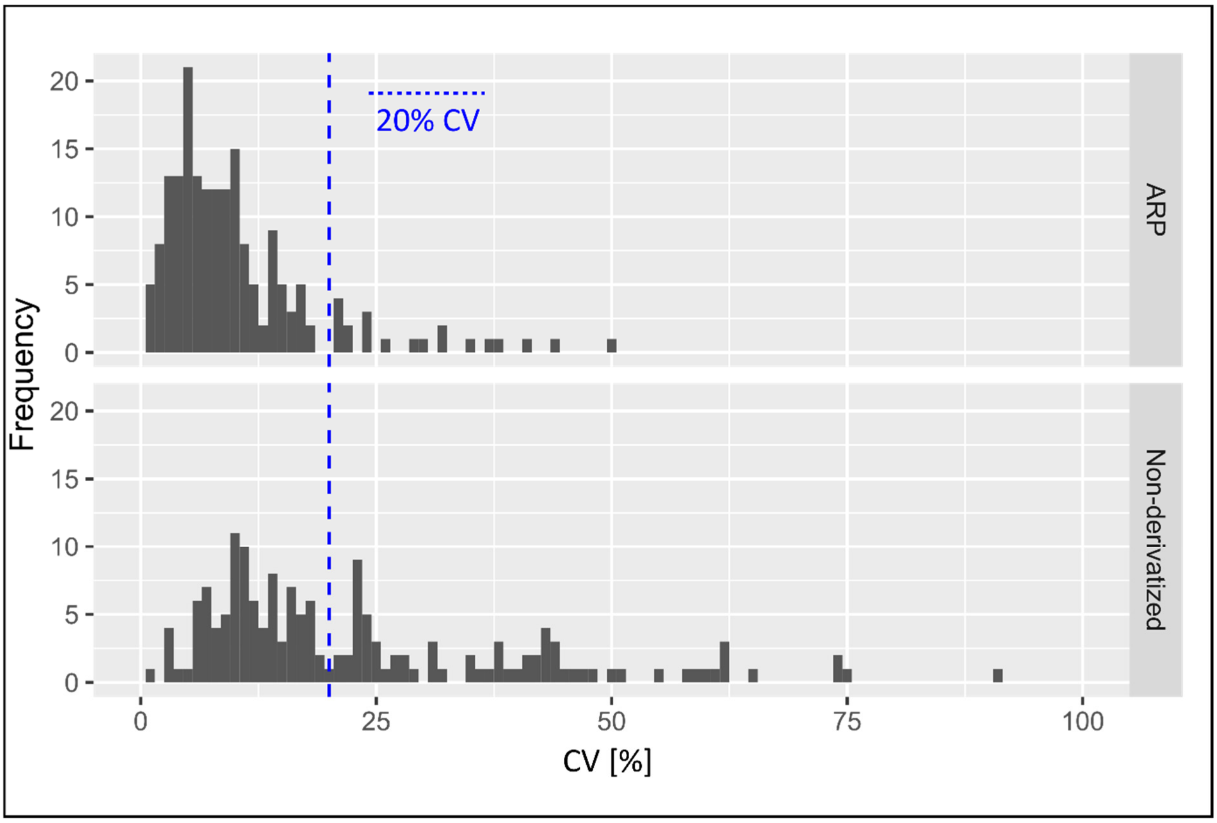

Figure S6. Histograms displaying percentage coefficient of variation (CV) of peak areas determined for ARP-peptides (top) and non-derivatized peptides (bottom) in ARP-Plasma:ARP-OxHSA samples. Blue dashed line corresponds to a 20% threshold.

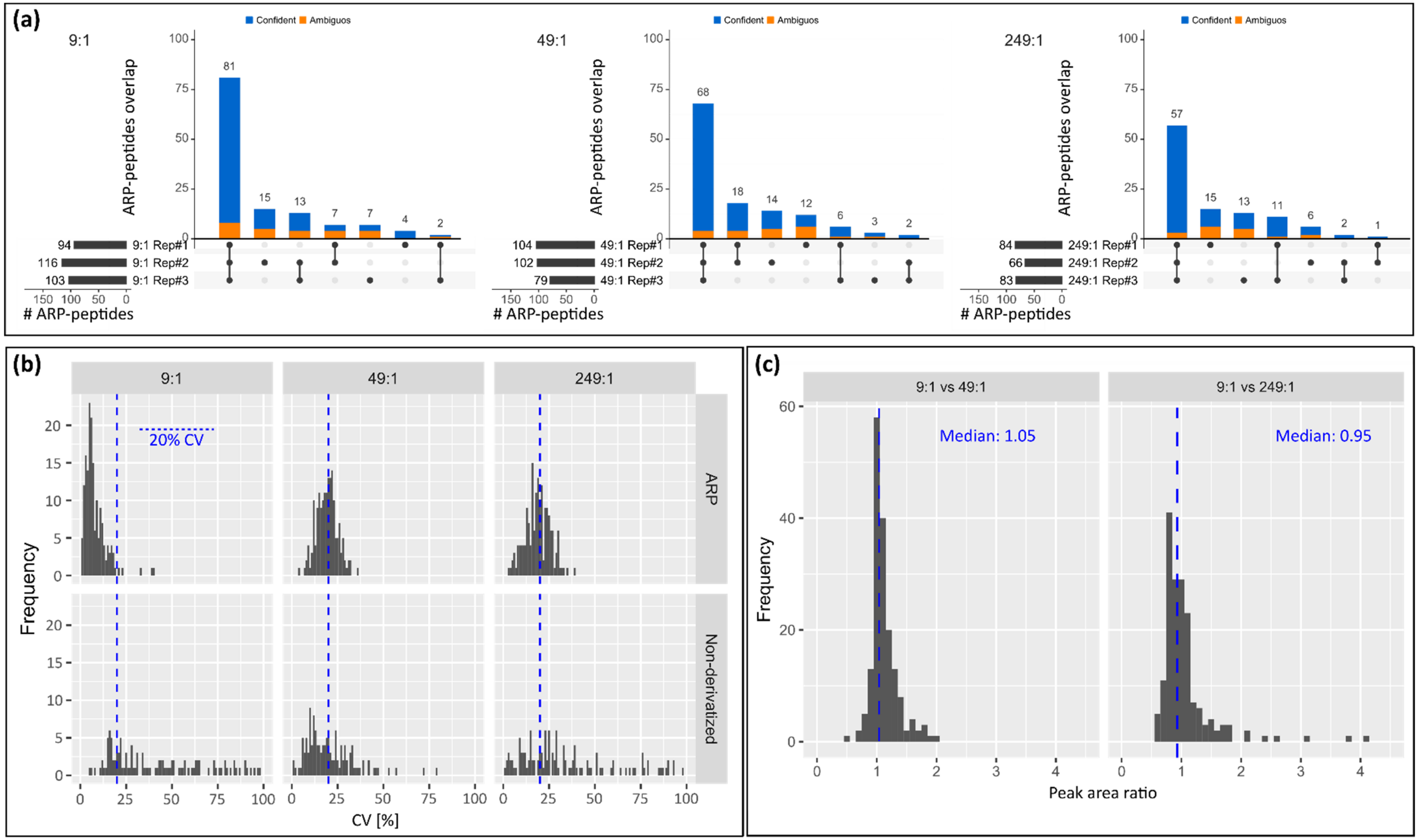

Figure S7. UpSet plots of ARP-peptides (**a**), histograms displaying coefficients of variation (CV) for peak areas of ARP-peptides and non-derivatized peptides (**b**), and histograms displaying peak area ratios of ARP-peptides between two sample mixtures (**c**). The vertical bars in the UpSet plots indicate the overlap in peptide identifications among the triplicates of each peptide mixture (i.e., 9:1, 49:1, and 249:1 Plasma:ARP-OxHSA), whereas the horizontal bars show the total number of ARP-peptide proposals for each replicate. The color code indicates the confidence level of peptide sequence and modification site identification (blue: confident, orange: ambiguous, and red: doubtful). Comparison of average peak area ratios of each ARP-peptide in the respective mixtures (9:1 vs. 49:1 and 9:1 vs. 249:1), the blue dashed lines indicates a ratio of 1 corresponding to equal signal responses.

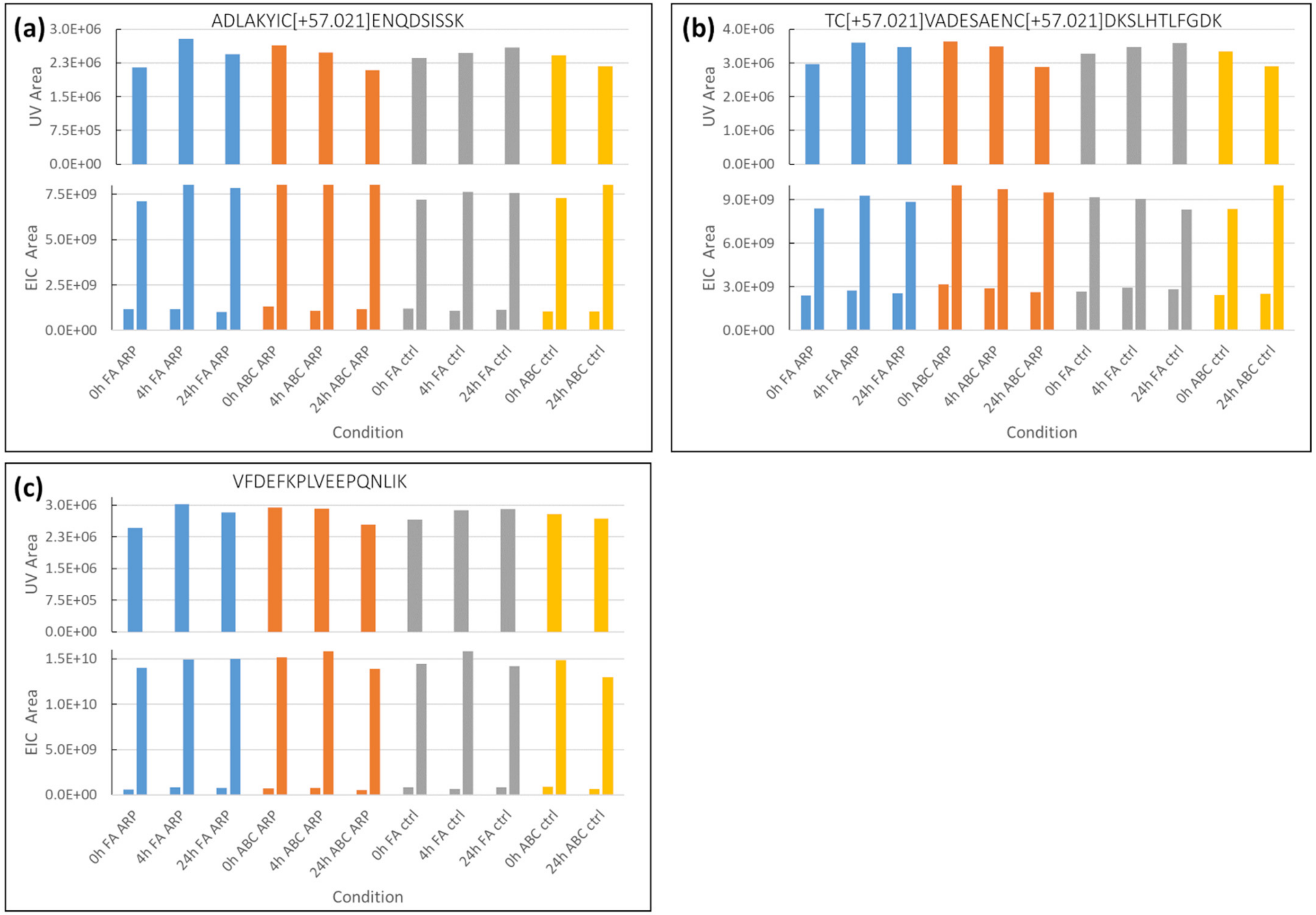

Figure S8. Stability of Gln/Asn and Glu/Asp-containing peptides in acidic conditions. A mix of three synthetic peptides corresponding to tryptic HSA peptide sequences were incubated in formic acid (1% (v/v)) and ammonium bicarbonate (25 mmol/L) with or without ARP (5 mmol/L). Each mixture was analyzed by RPC-MS after incubation periods of 0 h, 4 h, and 24 h. The absorbance was recorded at 214 nm and the EICs of the most intense peptide signals were monitored. Peak areas of the UV trace (top) and EICs (bottom) of signals detected at *m*/*z* 971.465 (z = 2) and 647.979 (z = 3) of peptide ADLAKYIC[+57.021]ENQDSISSK (panel (**a**)), at *m*/*z* 833.037 (z = 3) and 625.030 (z = 4) of peptide TC[+57.021]VADESAENC[+57.021]DKSLHTLFGDK. (panel (**b**), and at *m*/*z* 1023.051 (z = 2) and 682.370 (z = 3) of peptide VFDEFKPLVEEPQNLIK (panel (**c**)).
